# The Shadow Test

**Published:** 1890-12

**Authors:** 


*The Shadow Test.* By W. M. BEAUMONT. Pp. 38. London: H. K. LEWIS. 1890.

As far as it goes, this little book is good. It is another witness to the value of the Shadow Test in ophthalmic practice, and is written from the author's own experience.

The student will, however, find it wanting as a book of reference; and he is left entirely in the dark as to the optical conditions upon which the phenomena observed in this method of testing depend.

A book written upon this single subject is of little value unless it is exhaustive, which the present volume certainly is not.

We take this opportunity of pointing out that the direct method of estimating refractive error has the great advantage of giving practice to the observer, at the same time in seeing the details of the fundus of the eye, and in helping to make him at each estimation a better ophthalmologist; whereas the Shadow Test, though of undoubted value, especially in astigmatism, has no such combined advantage.

